# Urokinase Receptor uPAR Downregulation in Neuroblastoma Leads to Dormancy, Chemoresistance and Metastasis

**DOI:** 10.3390/cancers14040994

**Published:** 2022-02-16

**Authors:** Anna A. Shmakova, Polina S. Klimovich, Karina D. Rysenkova, Vladimir S. Popov, Anna S. Gorbunova, Anna A. Karpukhina, Maxim N. Karagyaur, Kseniya A. Rubina, Vsevolod A. Tkachuk, Ekaterina V. Semina

**Affiliations:** 1 National Cardiology Research Center of the Ministry of Health of the Russian Federation, Institute of Experimental Cardiology, 121552 Moscow, Russia; anyashm@gmail.com (A.A.S.); lex2050@mail.ru (P.S.K.); karina_ry@mail.ru (K.D.R.); tkachuk@fbm.msu.ru (V.A.T.); 2 Faculty of Medicine, Lomonosov Moscow State University, 119192 Moscow, Russia; galiantus@gmail.com (V.S.P.); gorbunovaanna94@gmail.com (A.S.G.); darth_max@mail.ru (M.N.K.); rkseniya@mail.ru (K.A.R.); 3 Koltzov Institute of Developmental Biology, Russian Academy of Science, 117334 Moscow, Russia; anna.karpukhina12@gmail.com

**Keywords:** urokinase receptor uPAR, neuroblastoma, Neuro2a, chemoresistance, metastasis, p53, cisplatin

## Abstract

**Simple Summary:**

uPAR is a membrane receptor that contributes to extracellular matrix remodeling and controls cellular adhesion, proliferation, survival, and migration. We demonstrate that the initially high uPAR expression predicts poor survival in neuroblastoma. However, relapsed neuroblastomas have a significantly decreased uPAR expression. uPAR downregulation in neuroblastoma cells leads to dormancy and resistance to chemotherapeutic drugs. In mice, low uPAR-expressing neuroblastoma cells formed smaller primary tumors but more frequent metastasis.

**Abstract:**

uPAR is a membrane receptor that binds extracellular protease urokinase, contributes to matrix remodeling and plays a crucial role in cellular adhesion, proliferation, survival, and migration. uPAR overexpression in tumor cells promotes mitogenesis, opening a prospective avenue for targeted therapy. However, uPAR targeting in cancer has potential risks. We have recently shown that uPAR downregulation in neuroblastoma promotes epithelial-mesenchymal transition (EMT), potentially associated with metastasis and chemoresistance. We used data mining to evaluate the role of uPAR expression in primary and relapsed human neuroblastomas. To model the decreased uPAR expression, we targeted uPAR using CRISPR/Cas9 and shRNA in neuroblastoma Neuro2a cells and evaluated their chemosensitivity in vitro as well as tumor growth and metastasis in vivo. We demonstrate that the initially high *PLAUR* expression predicts poor survival in human neuroblastoma. However, relapsed neuroblastomas have a significantly decreased *PLAUR* expression. uPAR targeting in neuroblastoma Neuro2a cells leads to p38 activation and an increased p21 expression (suggesting a dormant phenotype). The dormancy in neuroblastoma cells can be triggered by the disruption of uPAR-integrin interaction. uPAR-deficient cells are less sensitive to cisplatin and doxorubicin treatment and exhibit lower p53 activation. Finally, low uPAR-expressing Neuro2a cells formed smaller primary tumors, but more frequent metastasis in mice. To the best of our knowledge, this is the first study revealing the pathological role of dormant uPAR-deficient cancer cells having a chemoresistant and motile phenotype.

## 1. Introduction

The malignancy of cancer cells relies on several distinct biological capacities, known as “hallmarks of cancer”, which include invasion and metastasis [1]. Cancer cells are able to invade the surrounding tissues and distant organs via elevated production of proteolytic enzymes that degrade extracellular matrix (ECM) [2]. Urokinase-type plasminogen activator (urokinase, uPA) is an extracellular serine protease that cleaves plasminogen into active plasmin. In turn, plasmin plays an exceptionally significant role for cancer cell migration due to a large substrate specificity and its ability to activate matrix metalloproteases [3]. uPA binds its specific GPI-anchored receptor on the cell membrane (urokinase receptor, uPAR, encoded by the *PLAUR* gene) that promotes uPA activation and localizes extracellular proteolysis at the leading edge of migrating cells [4]. Given the ability of uPAR for lateral interaction with different membrane receptors (integrins, FPR, EGFR, etc.) resulting in intracellular signaling [5], the role of uPA/uPAR system extends far beyond simple coordination of proteolysis.

It is well documented that uPA and uPAR are overexpressed in various cancer types, although uPAR expression may be highly heterogeneous in a tumor cell population [6]. Overall their elevated expression is associated with poor clinical prognosis and increased mortality rate [7]. uPA and uPAR are commonly known to orchestrate cancer cell survival, proliferation, invasion and migration [8]. It was previously shown that uPAR downregulation decreases oncogenic potential of several cancer cell lines [9,10,11]. Despite the strong correlation between uPA system activation and tumor aggressiveness, uPA/uPAR targeting in cancer has not shown any convincing results in clinics [12]. Meanwhile new evidence emerges for a more complex and multifaceted role of uPAR in cell malignancy. Our recent data point to the dual role of uPAR in neuroblastoma: uPAR knockout decreases cell proliferation [9], but induces epithelial-mesenchymal transition (EMT) and promotes cell migration in vitro [13]. EMT is associated with aggressive features of cancer cells: an increased metastatic potential, cancer cell dormancy and chemoresistance [14,15]. To accelerate further research and therapeutic applications targeting uPAR in cancer therapy [8,16,17,18] and other pathologies [19], it is crucial to reveal the potentially negative consequences of uPAR silencing. 

Here we unmask a novel mechanism involving uPAR deficiency and leading to an increased cell malignancy (chemoresistance and metastasis). We demonstrate that uPAR downregulation in Neuro2a cells is associated with cell dormancy phenotype. The underlying mechanism is based on p38 activation and a decreased p53-mediated chemosensitivity. We have also shown that uPAR downregulation in neuroblastoma cells leads to a decreased primary tumor growth in contrast to more frequent metastasis. Thus, uPAR downregulation in tumors may potentially trigger the chemoresistant dormant metastasis.

## 2. Materials and Methods

### 2.1. Cell Culture

Mouse Neuro2a cells (ATCC^®^ CCL-131™) were chosen to analyze the role of uPAR in neuroblastoma in vitro and in vivo in mice. Neuro2a cells were cultured in complete medium: DMEM (Hyclone, Logan, UT, USA), 10% FBS, 1×MEM Non-Essential Amino Acids Solution, 1×antibiotic-antimycotic solution (all from Gibco, New York, NY, USA), 5% CO_2_, 37 °C. Cells were plated at a concentration of 1 × 10^5^ cells/mL.

Neuro2a cell clones with downregulated uPAR or complete uPAR knockout were obtained using CRISPR/Cas9 genome editing tool as described earlier [9,13,20,21]: clone #6 with complete uPAR knockout and clones #3 and #30 with significant uPAR suppression. The detailed procedure of uPAR suppression using a commercially-available plasmid vector encoding uPAR shRNA (Neuro2a-shuPAR) was published by us before [22]. The level of *Plaur* (uPAR) mRNA and protein expression in selected clones relative to wild-type Neuro2a cells (WT) is presented on Figure S1.

To evaluate Neuro2a cell signaling in response to the disruption of uPAR-integrin interaction, WT cells were seeded in six-well plates and treated with 100 nM α325 blocking peptide, 100 nM scrambled s325 peptide, or left untreated (control). After 24 h of treatment, cells were collected for western blot analysis. Blocking peptide α325 and control peptide s325 were synthesized at the Peptide Synthesis Laboratory of the National Cardiology Medical Center and characterized in our laboratory previously [23].

To evaluate the capacity of Neuro2a cells to metastatically colonize lungs in vivo, Neuro2a WT and Neuro2a-shuPAR cells were transfected with a plasmid pIRES containing the green fluorescent protein (GFP) as a reporter gene (pIRES-GFP). The cell line with stable GFP expression was obtained by culturing cells in a complete DMEM medium supplemented with 500 µg/mL G418 (Sigmaaldrich, St. Louis, MO, USA) for at least eight weeks. GFP expression was analyzed using a Leica DMI 6000 B fluorescent microscope and LAS X software. 

### 2.2. Analysis of Neuro2a Cell Sensitivity to Cisplatin and Doxorubicin

Cisplatin (CDDP) and doxorubicin were purchased from Teva (Israel) and diluted in a complete culture medium. For chemosensitivity analysis, Neuro2a cells were seeded at 70% confluence and treated with 30 μM CDDP. The CDDP working concentration was determined through comparing the cell apoptosis level in treated cells being significantly higher with that in non-treated control WT cells. The cells treated with PBS were used as a control for relative comparison. After 24 h of treatment, cells were collected for flow cytometry, western blot analysis and caspase activity assay.

The effect of doxorubicin on cell survival was analyzed using Neuro2a cells, which were seeded in a 96-well plate at a concentration of 1 × 10^4^ cells/well in 100 μL of culture medium and treated with 1 μM doxorubicin. MTT test was performed to evaluate cell viability before doxorubicin administration (0 h) and 24 h after as described earlier [24]. Briefly, at regular time points the cells were incubated with 0.1 mg of MTT (Merck Millipore, Burlington, MA, USA) for 2 h at 37 °C and then lysed in 100 μL of lysing buffer (25 mM HCl, 2% acetic acid, 3% DMF, 5% SDS, pH 4.7). The absorbance at 570 nm was measured using plate reader Infinite F200 PRO (Life Sciences, Tecan, Grodig, Austria).

### 2.3. Analysis of Neuro2a Cell Adhesion 

Neuro2a WT cells were plated into 24-well plates at a concentration of 2  ×  10^5^/well with the addition of 100 nM α325 blocking peptide, 100 nM scrambled s325 peptide, or nothing (control) to the medium. Cells were incubated in complete medium for 15 min, 30 min, 1 h or 2 h at 37 °C 5% CO_2_. Unattached cells were removed by a brief rinse with Hank’s balanced saline solution (HBSS) and a change of the medium. Images of attached cells in 3–5 equivalent fields of view for each well were captured using a time-lapse Leica phase-contrast light microscope (Leica AF6000 LX) equipped with a stage incubator with temperature (37 °C) and CO_2_ (5%) control, Leica DFC 350FX camera, and LAS AF software. The number of attached cells for each image was calculated using the ImageJ software. The experiment was performed in three biological replicates. 

### 2.4. Flow Cytometry

The level of apoptosis was analyzed using Annexin V—Cy5.5 (BD, cat. #559935) and propidium iodide staining according to manufacturer’s instructions. The fluorescence was measured using BD Accuri C6 Plus Flow Cytometer (BD Biosciences, San Jose, CA, USA). Annexin V-positive cells were considered apoptotic. Data were analyzed using FlowJo software (BD Biosciences), the same gating was applied to all samples within one experiment.

### 2.5. RNA Isolation, Reverse Transcription and qPCR

Total RNA was isolated by RNeasy^®^ Mini Kit (Qiagen, Valencia, CA, USA) following the manufacturer’s instructions. The quantity and quality of total RNA samples were measured using NanoDrop1000 spectrophotometer (Thermo Fisher Scientific, Waltham, MA, USA); 1% agarose gel with ethidium bromide was used to assess RNA integrity. 1.5 μg of total RNA was reverse-transcribed using oligo (dT) and random (dN)10 primers with MMLV RT kit (Evrogen, Moscow, Russia). PCR was carried out using qPCRmix-HS SYBR (Evrogen, Russia) on a CFX96™ Real-Time PCR Detection System (BioRad, Hercules, CA, USA). The murine cDNA primers were obtained from Evrogen and are listed in Table S1.

The thermal cycling program was as follows: a 5-min denaturing step at 95 °C followed by 40 amplification cycles consisting of 15 s denaturing at 95 °C, 15 s of annealing at 62 °C and 20 s of extension at 72 °C. qPCR reactions for each sample were performed in duplicates (technical replicates). A relative transcript level was calculated using the 2^−ΔΔCt^ method with *Actb* as a reference gene; for normalization the mean level of each transcript in wild-type cells (control) was taken as 1. 

### 2.6. Protein Extraction, Electrophoresis and Western Blot

Cells were harvested from culture dishes by cold cell scraper and centrifuged at +4 °C for 10 min at 500× *g*, supernatant was discarded, and cell pellet was washed with ice-cold phosphate-buffered saline (PBS, Sigma). PBS was then aspirated and cells were lysed in 50 μL of ice-cold RIPA lysis buffer (150 mM NaCl, 25 mM Tris-HCl, 0.5% sodium deoxycholate, 1% Nonidet P-40, 0.1% SDS, pH 7.4) [25] containing protease inhibitor cocktail (Thermo Scientific, Waltham, MA, USA) diluted 1:100. The lysates were vortexed 3 times during 20-min incubation on ice and centrifuged at +4 °C for 20 min at 16,000× *g*. The supernatant was transferred into a new pre-cooled microcentrifuge tube and the cell pellet was discarded. 5 μL of supernatant diluted 1:1000 was used for quantification of protein concentration by BCA assay (Thermo Scientific). Finally, after measuring the concentration, the lysates were dissolved in the equal volume of 2X Laemmli buffer with 10% β-mercaptoethanol and heated at 95 °C for 10 min.

Proteins (20 μg) were resolved in 10% SDS-PAGE gels and transferred to PVDF membrane (GE Healthcare, Chicago, IL, USA) in a transfer buffer (25 mM Tris, 192 mM glycine, 0.1% SDS and 20% methanol). PageRuler Prestained Protein Ladder (Thermo Scientific) was used as a molecular weight marker. Nonspecific binding was blocked in 5% non-fat dried milk, containing 0.1% Tween−20 at +4 °C overnight. Proteins were probed with the following primary antibodies (in 1:1000 dilution): rabbit anti-uPAR (cat. # sc-10815, Santa Cruz, Dallas, TX, USA), mouse anti-p53 (cat. # sc-98, Santa Cruz), rabbit anti-phospho-p53 (Ser15) (cat. # 9284, Cell Signaling, Danvers, MA, USA), mouse anti-p38α (cat. # 9217, Cell Signaling), rabbit anti-phospho-p38 (cat. # ab32557, Abcam, Abcam, Cambridge, MA, USA), rabbit anti-p21 (cat. # sc-397, Santa Cruz), rabbit anti-PARP1 (cat. # sc-7150, Santa Cruz), rabbit anti-caspase-3 (cat. # ab32351, Abcam), rabbit anti-γH2AX (cat. # 2577S, Cell signaling), rabbit anti-MDM2 (cat. #sc-812, Santa Cruz) mouse anti-β-actin (control of protein load, cat. # sc-81178, Santa Cruz), mouse anti-vinculin (control of protein load, cat. # V9131, Sigma-Aldrich) for 2 h at room temperature. First, the membranes were washed with PBS, containing 0.1% Tween-20, incubated with appropriate peroxidase-conjugated secondary antibodies (Imtek, Moscow, Russia) (in 1:10,000 dilution) at room temperature for 1.5 h, and washed in PBS afterwards (containing 0.1% Tween-20). Proteins were visualized using SuperSignal West Pico Chemiluminescent Substrate (Thermo Scientific) and ChemiDoc™ XRS + System (BioRad) for western blotting imaging and analysis. In each case a reproducible result out of at least three independent experiments is presented. Densitometric analysis of blots was performed in ImageJ (NIH, Bethesda, MD, USA). Uncropped western blot images are presented on Figure S4.

### 2.7. Caspase-3 Activity Measurement

Caspase-3 activity was measured as described earlier [26]. Briefly, caspase-3 activity was assessed by detecting the cleavage of fluorogenic peptide substrate DEVD-AMC (PeptaNova, Sandhausen, Germany). Harvested cells were resuspended in PBS supplemented with 0.5 mM PMSF and Roche complete protease inhibitors (100 μL PBS per 1 × 10^6^ cells). 25 μL of the suspension was placed into a 96-well plate and mixed with the peptide substrate (40 μM), and dissolved in 75 μL of caspase-3 reaction buffer (100 mM HEPES, pH 7.2, 10% sucrose, 5 mM DTT, 0.001% NP-40, 0.1% CHAPS). The cleavage of fluorogenic peptide was monitored at 37 °C using VarioScan Flash multimode detector (Thermo Scientific) by AMC liberation at 380 nm excitation and 460 nm emission. The fluorescence values were normalized to the protein concentrations measured using the Pierce BCA Protein Assay Kit (Thermo Scientific).

### 2.8. Animal Studies

Adult C57BL/6N wild-type (Pushchino, Pushchino, Russia) mice were enrolled in the study. Mice were kept in cages by three and maintained under a standard 12-h light cycle, with temperature 20–24 °C and humidity 35–70%. Water and food were available ad libitum. Animal housing was performed in accordance with European Convention for the Protection of Vertebrate Animals used for Experimental and other Scientific Purposes ETS №123. A careful consideration was given to the number of animals: a cohort of 17 animals (9 males and 8 females, 9–15 weeks) was used, the minimum number required to obtain valid results. Particular effort was made to minimize the animals’ pain and distress. For the following series of experiments, mice were randomly divided into two groups. The experimental procedures were conducted in accordance with the Directive 2010/63/EU of the European Parliament and the Council of 22 September 2010 on the protection of animals used for scientific purposes. All manipulations with animals were approved by the local ethical committee in accordance with the in-house requirements of the Commission on Bioethics of the Lomonosov Moscow State University (license number 3.2).

Mice were inoculated subcutaneously in the posterior flank with 10^6^ GFP-positive Neuro2a cells (WT (*n* = 7) or Neuro2a-shuPAR (*n* = 10)), premixed with 200 μL of ice-cold growth factor-reduced Matrigel^TM^ (Growth Factors reduced, Corning, NY, USA). 3 weeks after injection, tumor size was measured using calipers by the longest diameter, mice were lethally anesthetized by intraperitoneal injection of 400 μL of a 2.5% solution of 2,2,2-tribromoethanol (Sigmaaldrich), the lungs were isolated, frozen in TISSUE-TEK^®^ OCT COMPOUND and the analysis of the metastases of Neuro2a cells was performed. 

### 2.9. Analysis of Lung Metastasis 

Lungs is the common analyzed site of metastasis after subcutaneous injection of tumor cells in mice [27]. Cross sections of isolated mice lungs were obtained and then fixed in 4% paraformaldehyde (Panreac, Barcelona, Spain), washed in PBS, permeabilized with 0.1% Triton X-100 (Triton^®^X-100, Peroxide Free, Panreac) and washed in PBS. To block the non-specific binding, sections were treated with 10% normal donkey serum (Sigmaaldrich) for 1 h, washed in PBS and counterstained with DAPI Sigmaaldrich, 1:10,000. Samples were embedded in Aqua Poly Mount mounting medium (Polysciences, Warrington, PA, USA). Images of lung metastases were acquired using confocal laser scanning microscopy system Zeiss (LSM 780) and ZEN 2010 software. DAPI and GFP fluorescence were sequentially excited using lasers with 405 and 488 wave lengths, respectively. The number of GFP+ cells per image view was calculated using the ImageJ software (NIH). 

### 2.10. Mining Transcriptome Data for Neuroblastoma

The microarray-based gene expression data for the Versteeg cohort (NCBI GEO accession GSE16476 [28]) were downloaded and analyzed in the R2 database (http://r2.amc.nl, accessed on 12 July 2021). The microarray-based gene expression data for primary and relapsed neuroblastoma, published by Schramm et al. (NCBI GEO accession GSE65303 [29]), were downloaded and compared using the R2 database (http://r2.amc.nl, accessed on 12 July 2021). 

### 2.11. Data and Statistical Analysis

All quantitative data are presented as mean ± standard error of mean (SEM). Student’s unpaired *t*-test was applied for two-group comparisons. One-way analysis of variance (1-way ANOVA) was performed for multiple-group comparisons with one factor, two-way ANOVA (2-way ANOVA) was performed for multiple-group comparisons with two factors, both tests were followed by Dunnett’s post-hoc test (relative to WT or control group). The level of significance was set at *p* < 0.05. Data were analyzed using GraphPad Prism 9 (GraphPad Software Inc., San Diego, CA, USA).

## 3. Results

### 3.1. High Initial *PLAUR* Expression in Neuroblastoma Predicts Poor Survival; a Decrease in *PLAUR* Expression Is Associated with Tumor Relapse

**Figure 1:**
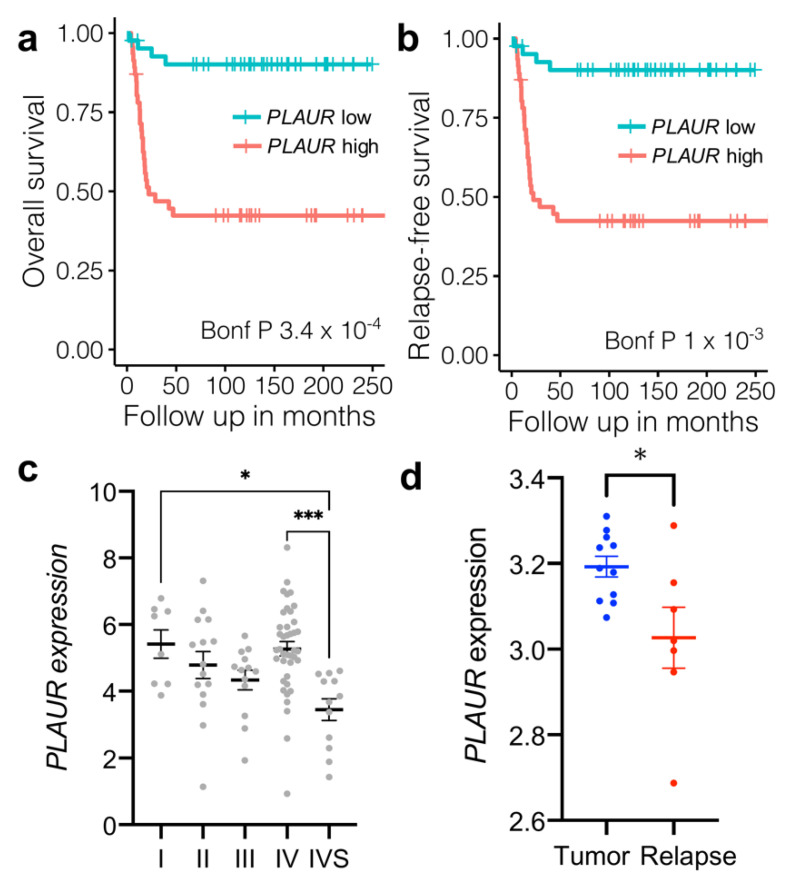
uPAR expression correlates with survival and relapse in human neuroblastoma. (**a**) Overall survival of human neuroblastoma patients stratified by low (red) and high (blue) expression of *PLAUR*. (**b**) Relapse-free survival of human neuroblastoma patients stratified by low (red) and high (blue) expression of *PLAUR*. Kaplan-Meier survival curves were generated from Versteeg cohort [28] data, NCBI GEO accession GSE16476, and compared using the R2 database (http://r2.amc.nl, accessed on 12 July 2021). (**c**) The log2 of *PLAUR* expression is shown in the I, II, III, IV and IVS stage neuroblastomas as classified by the International Neuroblastoma Staging System (INSS). The data were obtained from Versteeg cohort [28], NCBI GEO accession GSE16476, and compared using the R2 database (http://r2.amc.nl, accessed on 12 July 2021). 1-way ANOVA, Tukey’s post-hoc, * indicates *p* < 0.05, *** indicate *p* < 0.001. (**d**) The log2 of *PLAUR* expression is shown in the primary and relapsed tumors. The data were obtained from Schramm et al. [29], NCBI GEO accession GSE65303, and compared using R2 database (http://r2.amc.nl, accessed on 12 July 2021). * indicates *p* < 0.05.

First, we examined the role of uPAR in human neuroblastomas. We mined the microarray gene expression data of 88 cases of neuroblastoma (Versteeg cohort; [28]). All samples were obtained from primary tumors of untreated patients. We found that high *PLAUR* expression before treatment initiation was significantly associated with poor overall survival and shorter relapse-free survival period in human neuroblastoma patients (Figure 1a,b). Of note, the expression of uPA (*PLAU*) and its inhibitor PAI-1 (*SERPINE1*) were not significantly correlated with survival in this cohort (Figure S2). Surprisingly, uPAR expression did not increase with neuroblastoma’s stage (Figure 1c), as it was shown previously, for example, in the case of glioblastoma [30]. In fact, uPAR expression was lower at IVS stage as compared to stage I and IV (Figure 1c).

To address the role of uPAR in neuroblastoma progression after treatment, we further analyzed the dynamics of uPAR expression in microarray gene expression data based on 18 cases of primary and relapsed (locoregional or distant metastasis) neuroblastomas, published by Schramm et al. [29]. We found that the relapse of neuroblastoma after treatment is associated with the decrease in *PLAUR* expression (Figure 1d). 

These obtained data indicate that high *PLAUR* expression in neuroblastoma at the early stage is associated with worsened survival. However, uPAR content can be subject to changes upon tumor progression or treatment. Therefore, we hypothesized that the decrease in uPAR expression in relapsed tumors may have an impact on neuroblastoma malignancy and progression (chemoresistance, metastasis). To unravel the role uPAR plays in cancer progression, we next explored the effects and mechanisms of uPAR downregulation on neuroblastoma chemosensitivity.

### 3.2. uPAR Downregulation Is Associated with Chemoresistance in Neuroblastoma Neuro2a Cells

**Figure 2:**
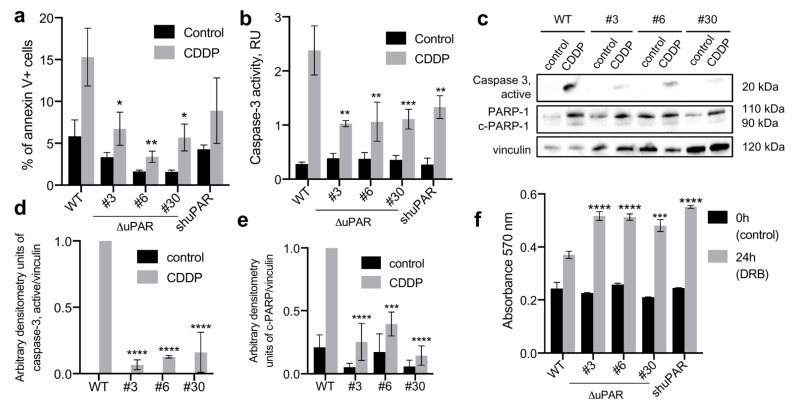
uPAR downregulation in Neuro2a cells is associated with chemoresistance. (A-G) Neuro2a cells with different uPAR expression were treated with cisplatin (CDDP) and collected 24 h later, non-treated cells were used as a control. (**a**) The percentage of Annexin V-positive (apoptotic) Neuro2a cells in control culture and after CDDP treatment. (**b**) Enzymatic caspase-3 activity in control cells and cells treated with CDDP. (**c**) Western blot analysis of caspase-3 activation and PARP-1 cleavage in Neuro2a cells in control and after CDDP treatment. Vinculin was used as a loading control. A reproducible result is presented. (**d**) Densitometry analysis of active caspase-3 normalized to vinculin. (**e**) Densitometry analysis of cleaved PARP-1 fragment normalized to vinculin. (**f**) Neuro2a cells with different uPAR expression were seeded in a 96-well plate and the MTT test was performed to evaluate cell viability before the addition of doxorubicin (0h) and 24 h after. The absorbance at 570 nm was measured. Data are presented as mean ± SEM, 2-way ANOVA, Dunnett’s post-hoc, * indicates *p* < 0.05; ** indicate *p* < 0.01; *** indicate *p* < 0.001; **** indicate *p* < 0.0001 compared to WT cells. WT, control Neuro2a cells; #3, #6, and #30, uPAR-deficient clones of Neuro2a cells; Neuro2a-shuPAR, cells transfected with shRNA to suppress uPAR; CDDP, cisplatin; DRB, doxorubicin; PARP, poly-ADP-ribose-polymerase.

The effect of cisplatin (CDDP) treatment was examined using neuroblastoma Neuro2a cells, where uPAR expression was targeted either by CRISPR/Cas9n (Neuro2a-ΔuPAR, clones #3, #6, #30) or by shRNA (Neuro2a-shuPAR) to avoid the effects related to the individual features of the selected clones. uPAR expression was almost negligible in Neuro2a-ΔuPAR clones and two-fold decreased in Neuro2a-shuPAR as compared to the wild-type (WT) Neuro2a cells (Figure S1). The cells were treated with CDDP for 24 h and the apoptosis activation was analyzed as described earlier (Figure 2). We found that upon CDDP treatment, the percentage of apoptotic (Annexin V+) cells was significantly lower in Neuro2a-ΔuPAR clones compared to WT: 6.7 ± 2.0% in clone #3, 3.4 ± 0.7% in clone #6, 5.7 ± 1.6% in clone #30 vs 15.3 ± 3.5% in WT cells (2-way ANOVA, Dunnett’s post-hoc, *p* = 0.0456, *p* = 0.0054, *p* = 0.0237, respectively, Figure 2a). A non-significant decrease in the percentage of apoptotic cells was observed in Neuro2a-shuPAR cells (8.9 ± 3.9%). No significant changes in the percentage of apoptotic cells without CDDP treatment (control) were detected.

We next analyzed the enzymatic activity of caspase-3, a crucial mediator of apoptotic cell death [31], in cell lysates both after CDDP treatment and without treatment. The caspase-3 activation appeared to be significantly lower in both Neuro2a-ΔuPAR clones and Neuro2a-shuPAR cells upon CDDP treatment: 1.03 ± 0.06 in #3 clone, 1.06 ± 0.36 in #6 clone, 1.11 ± 0.18 in #30 clone, 1.33 ± 0.21 in Neuro2a-shuPAR vs 2.38 ± 0.45 relative units in WT cells (2-way ANOVA, Dunnett’s post-hoc, *p* = 0.0067, *p* = 0.0083, *p* = 0.0008, *p* = 0.0089, respectively, Figure 2b). No significant changes in the caspase-3 enzymatic activity were detected in cells without CDDP treatment (control).

We also examined the content of caspase-3 active form and PARP-1 using Western blotting. As expected, lower caspase-3 activation was observed in Neuro2a-ΔuPAR clones compared to WT upon CDDP treatment (Figure 2c,d). The PARP-1 cleavage (c-PARP-1), another hallmark of caspase-mediated apoptosis [32], was also decreased in Neuro2a-ΔuPAR clones compared to control (Figure 2c,e). 

These data clearly demonstrate that CDDP treatment of uPAR-deficient Neuro2a cells results in a significantly lower apoptosis activation, pointing to their increased resistance to chemotherapy as compared to WT cells. These findings were further confirmed by an increased survival of uPAR-deficient cells upon doxorubicin treatment (Figure 2f). Hence, uPAR downregulation in Neuro2a cells is associated with chemoresistance.

### 3.3. uPAR-Deficient Neuro2a Cells Exhibit Dormant Phenotype and Decreased p53 Activation upon CDDP Treatment

**Figure 3:**
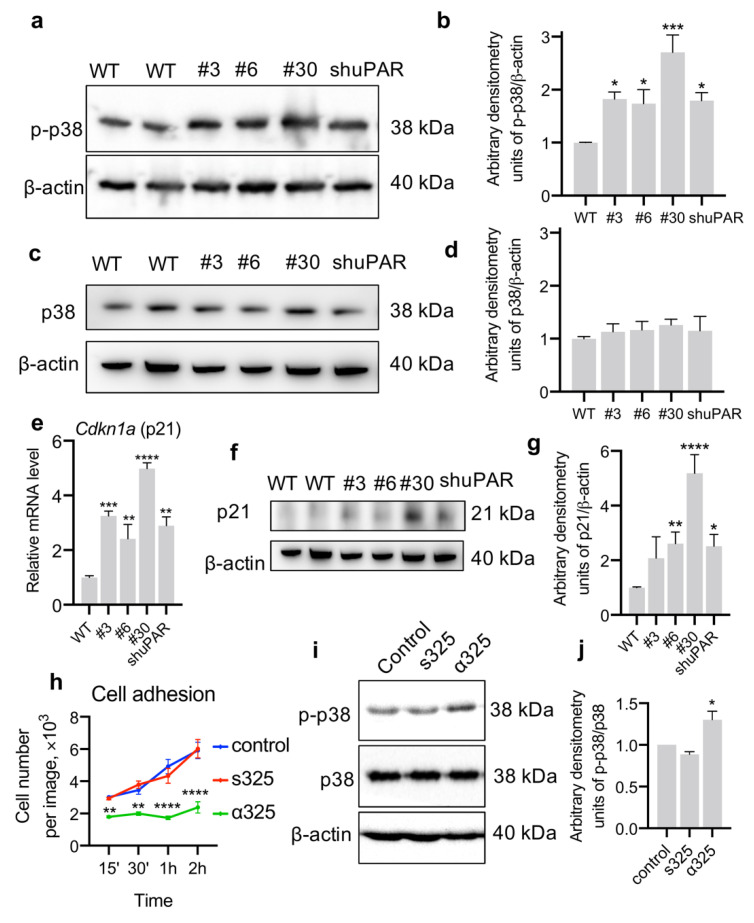
uPAR downregulation leads to dormant phenotype of Neuro2a cells. (**a**) Western blot analysis of p38 phosphorylation in Neuro2a cells. β-actin was used as a loading control. A reproducible result is presented. (**b**) Densitometry analysis of p38 phosphorylation normalized to β-actin. (**c**) Western blot analysis of total p38 content in Neuro2a cells. β-actin was used as loading control. A reproducible result is presented. (**d**) Densitometry analysis of total p38 content normalized to β-actin. (**e**) *Cdkn1a* (p21) mRNA expression in Neuro2a cells with different uPAR expression as analyzed by qPCR. The mRNA level was normalized to Actb expression as a housekeeping gene, the normalization was performed assuming the mean level of transcript in WT cells as 1. (**f**) Western blot analysis of p21 content in Neuro2a cells. β-actin was used as a loading control. A reproducible result is presented. (**g**) Densitometry analysis of p21 content normalized to β-actin. (**h**) Neuro2a cells were seeded into cell culture plates and treated with α325, peptide that blocks α3/α5β1-integrin-uPAR interaction, scrambled peptide s325 or left untreated (control). The medium with non-adherent cells was replaced 15′, 30′, 1 h or 2 h later and cell adhesion was analyzed using light microscopy. Graph shows the mean number of attached cells calculated at least in three fields of view in three different wells. (**i**) Western blot analysis of p38 phosphorylation and total p38 content in Neuro2a WT cells treated for 24 h with α325, peptide that blocks α3/α5β1-integrin-uPAR interaction, scrambled peptide s325 or left untreated (control). β-actin was used as a loading control. A reproducible result is presented. (**j**) Densitometry analysis of p38 phosphorylation normalized to total p38. Data are presented as the mean ± SEM. 1-way (**b**,**d**,**e**,**g**,**j**) or 2-way (**h**) ANOVA, Dunnett’s post-hoc, * indicates *p* < 0.05, ** indicate *p* < 0.01, *** indicate *p* < 0.001, **** indicate *p* < 0.0001 compared to WT or control (untreated) cells. WT, control Neuro2a cells; #3, #6, and #30, uPAR-deficient clones of Neuro2a cells; Neuro2a-shuPAR, cells transfected with shRNA to suppress uPAR.

One of the mechanisms that renders cancer cells insensitive to chemotherapy is cellular dormancy, or the quiescent state. Cancer cell dormancy is characterized by a reversible mitotic and growth arrest, lack of proliferative signaling and resistance to apoptosis [33,34,35]. We have previously shown that uPAR-deficient Neuro2a cells have a decreased proliferative potential [9] via a reduced activity of mitogen-activated protein kinase ERK1/2 (extracellular signal-regulated kinase) [13]. To further untangle the signaling pathways responsible for chemoresistance in uPAR-deficient cells, we analyzed the activation of p38 (a benchmark of cancer cell dormancy) [33]. p38 phosphorylation was increased by more than 1.5-folds in Neuro2a-ΔuPAR clones #3 and #6 compared to WT cells (Figure 3a,b) without significant changes in p38 total content (Figure 3c,d). p21 (*Cdkn1a*) is a downstream effector of p38 signaling and a negative regulator of cell cycle, being another molecular benchmark of dormancy [33,34,35]. To verify the dormant phenotype of uPAR-deficient Neuro2a cells, we evaluated *Cdkn1a* mRNA expression in Neuro2a cells and detected its significant increase in Neuro2a-ΔuPAR clones (3.25 ± 0.24 in clone #3, 2.42 ± 1.04 in clone #6, 4.99 ± 0.37 in clone #30) and Neuro2a-shuPAR cells (2.90 ± 0.45) vs WT cells (1.00 ± 0.17) (1-way ANOVA, Dunnett’s post-hoc, *p* = 0.0008, *p* = 0.0046, *p* < 0.0001, *p* = 0.0033, respectively, Figure 3e). This was further confirmed by an increase of p21 expression on the protein level in Neuro2a-ΔuPAR clones and Neuro2a-shuPAR cells compared to WT cells (Figure 3f,g).

It was previously shown that disassembly of uPAR-integrin complexes correlates with p38 activation and dormancy in epidermoid carcinoma cells [36]. We hypothesized that p38 activation in uPAR-deficient neuroblastoma cells is caused by decreased uPAR-integrin interaction. In order to prove that, we incubated Neuro2a WT cells with α325, a short peptide that selectively blocks the interaction of α3/α5β1-integrin with uPAR [23,37]; cells treated with scrambled peptide s325 or non-treated cells were used as a control. The activity of α325 in Neuro2a WT cells was confirmed by the analysis of cell adhesion: the addition of α325 to the cell medium significantly decreased Neuro2a adhesion (Figure 3h), which is consistent with previous reports in other cell lines [37]. We next analyzed the activation of p38 and found that p38 phosphorylation was 1.3-fold higher in Neuro2a WT cells treated with α325 compared to non-treated and s325-treated cells without significant changes in p38 total content (Figure 3i,j). The fact that α325 peptide selectively blocks uPAR interaction with α3/a5β1-integrin, but not other integrins and membrane receptors, can explain why p38 activation is not so prominent in the case of α325 treatment compared to uPAR downregulation. It nevertheless confirms that the disruption of uPAR-integrin interaction can lead to p38 activation.

**Figure 4:**
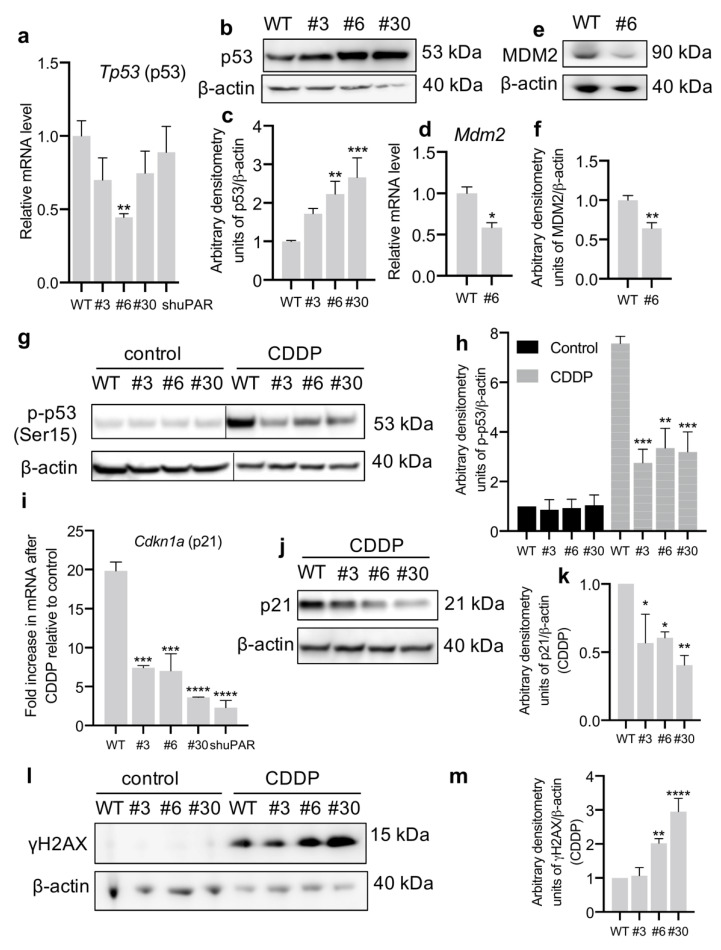
uPAR downregulation results in decreased p53 activity in Neuro2a cells treated with CDDP. (**a**) *Tp53* mRNA expression in Neuro2a cells with different level of uPAR expression as tested by qPCR. The mRNA level was normalized to *Actb* expression as a housekeeping gene, the normalization was carried out assuming as 1 the mean level of transcript in WT cells. (**b**) Western blot analysis of total p53 content in Neuro2a cells. β-actin was used as loading control. A reproducible result is presented. (**c**) Densitometry analysis of total p53 content normalized to β-actin. (**d**) *Mdm2* mRNA expression in Neuro2a cells as analyzed by qPCR. The mRNA level was normalized to *Actb* expression as a housekeeping gene, assuming the mean level of transcript in WT cells as 1. (**e**) Western blot analysis of MDM2 content in Neuro2a WT and #6 cells. β-actin was used as loading control. A reproducible result is presented. (**f**) Densitometry analysis of MDM2 content normalized to β-actin. (**g**) Western blot analysis of p53 phosphorylation on Ser15 in Neuro2a cells (in control conditions and after 24 h of CDDP treatment). β-actin was used as a loading control. A reproducible result is presented. The black lines indicate where the lanes were cut from the same immunoblot image and pieced together to allow for a direct comparison. (**h**) Densitometry analysis of p53 phosphorylation normalized to β-actin. (**i**) Fold increase in *Cdkn1a* mRNA expression in Neuro2a cells expression after 24 h of CDDP treatment compared to control (non-treated) cells as analyzed by qPCR. The mRNA level was normalized to *Actb* expression as a housekeeping gene, the normalization was carried out assuming the mean level of transcript in control (non-treated) WT cells as 1. The fold increase was calculated as ratio of p21 expression level in CDDP-treated cells to the respective control for each cell type. (**j**) Western blot analysis of p21 content in Neuro2a cells after 24 h of CDDP treatment. β-actin was used as loading control. A reproducible result is presented. (**k**) Densitometry analysis of p21 content normalized to β-actin. (**l**) Western blot analysis of γH2AX in Neuro2a cells (in control conditions and after 24 h of CDDP treatment). β-actin was used as a loading control. A reproducible result is presented. (**m**) Densitometry analysis of γH2AX content normalized to β-actin. Data are presented as the mean ± SEM. *t*-test (**d,f**), 1-way (**a,c,i,k,m**) or 2-way (**h**) ANOVA, Dunnett’s post-hoc, * indicates *p* < 0.05, ** indicate *p* < 0.01, *** indicate *p* < 0.001, **** indicate *p* < 0.0001 compared to WT cells. WT, control Neuro2a cells; #3, #6, and #30, uPAR-deficient clones of Neuro2a cells; Neuro2a-shuPAR, cells transfected with shRNA to suppress uPAR; CDDP, cisplatin.

The signaling mechanisms regulating cell dormancy and signaling via p38 are intertwined with p53 activity, a key tumor suppressor and regulator of cellular response to DNA damage induced by chemotherapy [38,39]. To test whether uPAR downregulation impacts the p53 axis, first we analyzed the expression of *Tp53* mRNA. We found that the complete uPAR knockout (clone #6) led to a ~2-fold decrease in *Tp53* mRNA expression: 0.44 ± 0.03 in #6 vs 1.00 ± 0.10 in WT (1-way ANOVA, Dunnett’s post-hoc, *p* = 0.0062) (Figure 4a). Surprisingly, in contrast to decreased mRNA, p53 protein level was much higher in all Neuro2a-ΔuPAR cell types (#3, #6, #30) compared to control (Figure 4b,c). This discrepancy may be explained by a high p53 protein stability. In order to explain the increased p53 protein content in clone #6 with decreased *Tp53* mRNA, we evaluated MDM2 expression, since p53 level is tightly controlled by MDM2, a p53-specific E3 ubiquitin ligase [39]. We found that *Mdm2* mRNA expression was, indeed, decreased in clone #6 (0.58 ± 0.06 in #6 vs 1.00 ± 0.08 in WT, *t*-test, *p* = 0.0135, Figure 4d). As anticipated, the level of MDM2 protein was also lower in clone #6 (Figure 4e,f), potentially accounting for the increased p53 stability.

At the next stage we tested whether the p53 accumulation affected its activity in Neuro2a-ΔuPAR cells. Hence, we estimated p53 activation by Ser15 phosphorylation [40] after 24 h of CDDP treatment (Figure 4g,h). We found that p53 activation in response to CDDP was much lower in Neuro2a-ΔuPAR cells compared to WT cell, which correlates with the data on resistance to CDDP (Figure 2). This was further confirmed by expression analysis of p21 (one of the p53 direct transcriptional targets) [40]. Indeed, both p21 mRNA and protein level were lower in CDDP-treated Neuro2a-ΔuPAR cells compared to non-treated cells (Figure 4i–k). We also evaluated the presence of γH2AX, a marker of DNA damage that is progressively removed when p53 is active [41,42]. An accumulation of γH2AX was clearly seen in Neuro2a-ΔuPAR clones #6 and #30 compared to WT (Figure 4l,m). Thus, p53 activation upon CDDP treatment is decreased in Neuro2a-ΔuPAR cells, which is in agreement with their chemoresistance. 

### 3.4. uPAR-Deficient Neuroblastoma Cells Have an Increased Metastatic Potential 

**Figure 5:**
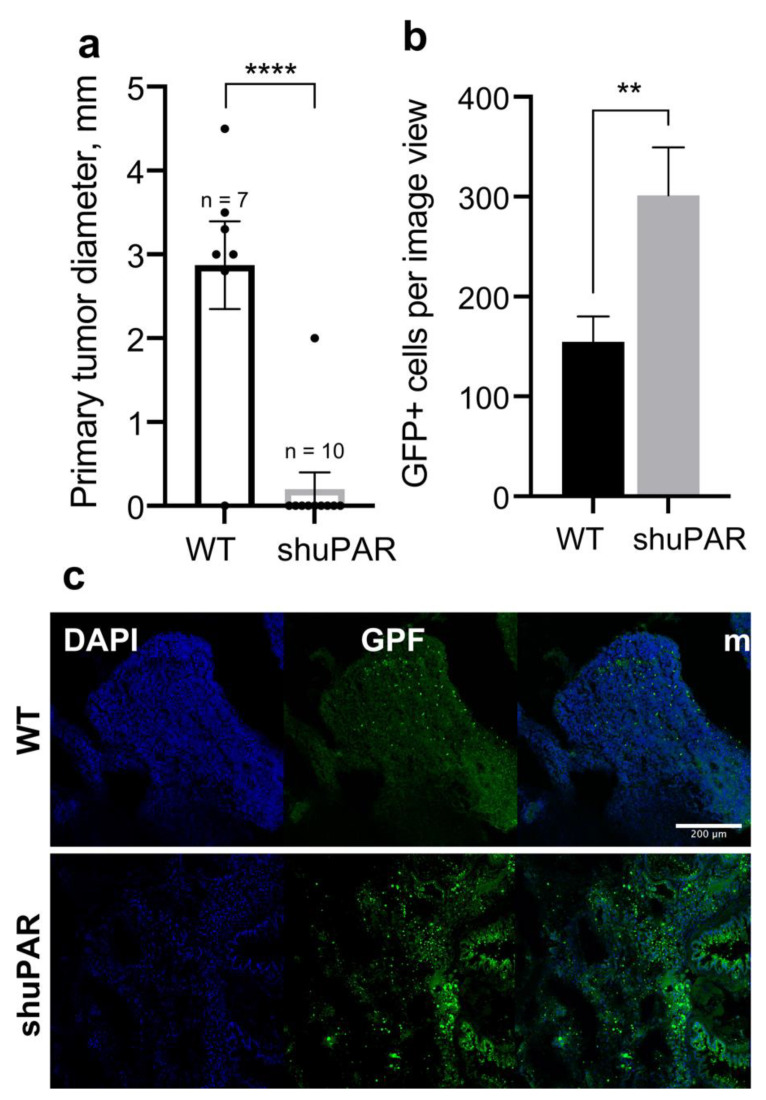
The role of uPAR in tumor growth and metastasis in vivo. (**a**) Mice were inoculated subcutaneously in the posterior flank with 10^6^ GFP-positive Neuro2A cells (WT or Neuro2a-shuPAR); tumor size was measured 3 weeks after injection. Data are presented as individual values, mean ± SEM. *t*-test, ****—*p* < 0.0001. (**b**) Three weeks after Neuro2a cell injection, mice were sacrificed and lung tissue sections were analyzed for the presence of GFP-positive cells. The number of GFP positive cells per image was evaluated. Data are presented as mean ± SEM. *t*-test, **—*p* < 0.01. (**c**) Representative images of lung tissue sections in mice 3 weeks after Neuro2a cell injection, DAPI was used for nuclear staining. Scale bar 200 µm.

Cancer cell dormancy and EMT are interconnected and have a high pathological significance for metastatic spread [35]. We have previously shown that uPAR-deficient Neuro2a cells exhibit a decreased proliferation but an increased migratory ability in vitro which correlates with an elevated expression of EMT transcription factor Snail [9,13]. To address the role of uPAR in neuroblastoma growth and metastasis in vivo, we mixed Neuro2a WT or Neuro2a-shuPAR cells, both expressing GFP as a reporter, with Matrigel and injected the mixture subcutaneously into mice to evaluate the tumor growth and lung metastasis three weeks later (Figure 5). We found that uPAR downregulation in neuroblastoma significantly inhibited the growth of primary tumors in vivo (*t*-test, *p* < 0.0001) (Figure 5a). We further evaluated the metastatic colonization of lungs by GFP-marked Neuro2a cells (Figure 5b,c). The number of GFP positive cells per image was calculated and we demonstrated that the number of Neuro2a-shuPAR cells was significantly higher than Neuro2 WT cells (*t*-test, *p* = 0.0061) (Figure 5b). Representative images of tissue sections are presented in Figure 5c. No significant difference in *Plau* expression could be detected in Neuro2a WT cells as compared to Neuro2a-shuPAR cells (Figure S3). These data underscore the importance of uPAR expression for primary tumor growth, while uPAR downregulation promotes metastatic spread.

## 4. Discussion

The wide diversity of the uPAR interactome is explicated by its numerous structurally and functionally different proteins. The interactome encompasses soluble ligands such as urokinase uPA, vitronectin, factor XII, SRPX2 and uPAR lateral membrane-bound partners including growth factor receptors (i.e., EGFR), G protein-coupled receptors, and integrins [5]. As a functional component of the plasminogen activator system, uPAR orchestrates ECM remodeling, cellular adhesion and migration [4,43]. Being a specific uPA receptor, uPAR is involved in proteolytic activation of growth factors and adhesion molecules, and plays a profound role in cell proliferation and survival [16]. In tumor cells, uPAR overexpression promotes mitogenesis via uPAR signalosome assembly, comprising integrins and growth factor receptors, which activity triggers ERK signaling and its downstream target proteins, and inhibits p38 pathway [44,45,46]. Accordingly, uPAR downregulation or inhibition has been shown to cause growth arrest, apoptosis, decreased cell survival and reduced metastatic spread in various types of tumors [10,47,48]. Our previously obtained results demonstrate that uPAR knockout inhibits EGFR/ERK signaling axis and Akt activation and leads to attenuated cell proliferation in neuroblastoma [9,49]. 

Li et al. have previously shown that double uPA+ and uPAR+ positivity in neuroblastoma is associated with worsened survival. However, it was not clear whether ligand or receptor expression by tumor cells contributed to this effect [50]. Our findings indicate that the initially high *PLAUR* (Figure 1a,b), but not *PLAU*, nor *SERPINE1* expression (Figure S2) predicts poor survival in human neuroblastoma, although no correlation with neuroblastoma stage has been observed (Figure 1c). Yet, the relapsed neuroblastomas exhibit a marked decrease in *PLAUR* expression (Figure 1d), thus indicating that uPAR takes part in neuroblastoma progression. Therefore, albeit promising to yield new therapeutic strategies in oncology, uPAR targeting in cancer cells might have potential risks.

In the current study, we propose and discuss a putative mechanism whereby neuroblastoma cells can overcome the growth inhibitory effect of uPAR downregulation and acquire a dormant, chemoresistant, and motile phenotype. We demonstrate that uPAR downregulation via either CRISPR/Cas9n or shRNA results in p38 activation and an increased p21 expression in Neuro2a cells (Figure 3). Alongside with downregulated ERK signaling, activated p38 and upregulated p21 are the well-known hallmarks of cancer cell dormancy [33,34,35]. As reported earlier, uPAR expression can be significantly lowered in dormant cells of different cancer types [45,51], and for head and neck carcinoma cells uPAR downregulation itself can induce dormancy [52,53]. Our data indicate that uPAR deficiency in neuroblastoma cells leads to a decrease in ERK signaling [9,13,49] and activation of p38 pathway (Figure 3). 

**Figure 6:**
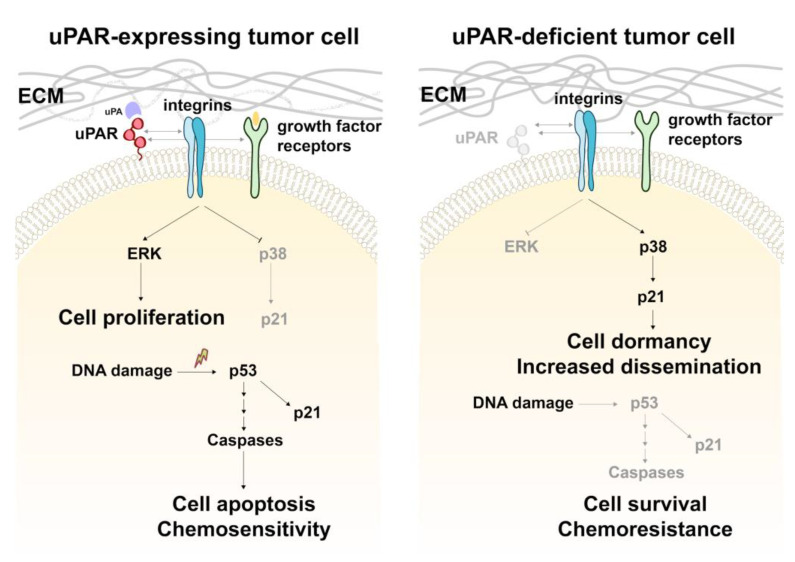
Putative mechanism of uPAR-mediated cell status regulation in neuroblastoma. Signalosome assembly comprising uPAR, integrins, growth factor receptors and their ligands on the plasma membrane can activate ERK signaling and inhibition of p38 pathway, which promotes cellular proliferation. In uPAR-expressing cells, DNA damage (e.g., by chemotherapeutic drugs) initiates p53 signaling, activates apoptosis overall making these cells susceptible to chemotherapeutic drugs. uPAR downregulation reverses the ERK/p38 ratio, triggering the activation of p38 signaling and resulting in reduced cellular proliferation and cell dormancy. uPAR-deficient dormant cells can evade the growth inhibitory and apoptotic stimuli. p53-mediated DNA damage response is diminished in these cells rendering their drug chemoresistance properties. uPAR-deficient cells are more prone to migration and dissemination. ECM, extracellular matrix; uPA, urokinase; uPAR, urokinase receptors.

The earlier published papers make an excellent job of detailing how uPAR can operate as a molecular switch in integrin signaling via the preferential activation of either ERK or p38 signaling pathway [5,36]. Our present study bears out earlier published data [35,45,54] and suggests that due to a decreased uPAR-integrin interaction on the plasma membrane in uPAR-deficient cells the ERK/p38 activity ratio is significantly declined (ERK^low^/p38^high^) leading to a loss of integrin-mediated mitogenic stimuli from ECM and resulting in a dormant phenotype (Figure 6). This is further confirmed by the use α325, a short peptide that selectively blocks the interaction of α3/α5β1-integrin with uPAR. We observed that Neuro2a treatment with α325 results in p38 activation (Figure 3i,j), which reinforces our conclusion that the disruption of uPAR-integrin interaction can lead to cell dormancy.

One of the main risks, associated with cancer cell dormancy is the loss of chemotherapy sensitivity. In the present study we demonstrate that uPAR-deficient cells are less sensitive to CDDP and doxorubicin treatment (Figure 2) and exhibit a lower level of p53 activation in response to chemotherapeutic drugs compared to WT cells (Figure 4). These results stand in drastic opposition to the well-established conceptualization of uPAR role in carcinogenesis. As reported earlier, uPAR expression has been associated with chemoresistance of several cancer cell types [55,56], while uPAR inhibition attenuated chemoresistance in multidrug resistant types of cancer lines, such as colon, cervical, head and neck cancer cell lines [10,56]. The underlying mechanism resides in uPAR-mediated lateral interaction with growth factor receptors resulting in ERK^high^-mediated pro-survival and proliferative signals received by uPAR-expressing cells even in conditions of withdrawal from growth-factor receptor signaling [57,58,59]. In contrast, recent studies indicate that dormant cells with decreased uPAR expression and low ERK activation, can exhibit an enhanced drug resistance [51]. Therefore, cancer cells may vary in their dependence upon proliferative signals transduced by the surface receptors and ECM: in certain cell types uPAR downregulation can trigger cell death, while in the others it can reduce cell proliferation rate and induce dormancy. The dormant cells in their turn can display resistance to apoptosis and anticancer therapy (Figure 6). This heterogeneity of cancer cells substantially complicates harnessing uPAR regulation as a therapy target for cancer treatment. 

Dormant cancer cells are also considered to be a major source of metastasis due to their insensitivity to treatment and decreased immune recognition [33]. Using an in vivo model of mouse neuroblastoma cells injected into mice, we found that uPAR downregulation significantly decreased the primary tumor growth (Figure 5a), which is consistent with the role of uPAR in sustaining cell proliferation. However, uPAR downregulation was associated with increased lung metastatic colonization in mice (Figure 5b,c). Similar data have been described for several cancer types that have limited primary tumor growth, yet demonstrate an active metastatic spread (e.g., carcinoma of unknown primary origin [60]). Moreover, recurrent human neuroblastomas, that frequently relapse with metastatic outgrowth, display a lowered expression of uPAR (Figure 1d). The increased metastatic spread exhibited by uPAR-deficient cells may be linked to their reduced cell adhesion to ECM. Actually, uPAR-integrin interaction promotes cellular adhesion, while the disruption of this interface or uPAR downregulation significantly inhibits cellular adhesion [13,61]. Accordingly, ablation of β1-integrin functioning can cause cellular senescence and increased cell dissemination of pancreatic β cell tumors [62]. Aligning with these data, we have previously demonstrated that uPAR downregulation in neuroblastoma cells results in EMT and increased migratory capabilities [13]. Particularly, when uPAR is downregulated, its unbound ligand uPA can be transported into the nucleus, where it operates as an activator of transcription factors—NF-κB and Snail, a key EMT transcription factor. uPAR-deficient neuroblastoma cells exhibit mesenchymal phenotype: increased migration capability, reduced adhesion, elevated expression of N-cadherin and decreased E-cadherin [13]. Using CRISPR/Cas9 method to knockout *PLAUR* gene in melanoma and colon cancer cell lines, Biagioni et al. observed reduced proliferation in uPAR-deficient cancer cell cultures coupled with a stem-like phenotype [63]. Taking into consideration the fact that EMT and stemness are risk factors of unfavorable outcome, the role of uPAR in tumor initial growth and cancer progression should be reconsidered. Towards that end, despite the fact that uPAR suppression or knockout results in a decreased tumor growth [10,47,48], uPAR downregulation can lead to tumor adaptation and render the uPAR targeting approach ineffective. These data reshapes the conceptual landscape of cancer biology illustrating the antagonistic functional duality of “cancer” genes [64] as illustrated by uPAR adverse functions. However, the solutions may come from combination approaches that target uPAR concomitantly with uPA, MMP, cathepsin B and other molecules to increase cancer therapy sensitivity and suppress tumor growth and metastasis [65,66,67].

## 5. Conclusions

Our current findings yield a highly intricate molecular hub with uPAR being a two-faced evil at its nexus. For the first time, we demonstrate that downregulated uPAR expression in neuroblastoma cells correlates with the induction of dormancy, chemoresistance, and metastatic spread. uPAR is regularly considered as a promising target for cancer prevention and treatment. However, our results reveal a much more complicated role of uPAR in defining cancer cell phenotype, signaling pathways, and overall tumor growth and metastasis. Therefore, the mechanisms underlying the action of uPAR in cancer progression should be carefully reconsidered. To the best of our knowledge, this is the first study elucidating the pathological significance of uPAR-deficient dormant cells.

## Data Availability

Publicly available datasets were analyzed in this study. This data can be found here: NCBI GEO accession GSE16476, GSE65303.

## Supplementary Materials

The following supporting information can be downloaded at: https://www.mdpi.com/article/10.3390/cancers14040994/s1. Figure S1: *Plaur* (uPAR) expression in Neuro2a cells analyzed by RT-qPCR and western blot; Figure S2: Overall and relapse-free survival of human neuroblastoma patients stratified by uPA and PAI-1 expression; Figure S3: *Plau* (uPA) mRNA expression in Neuro2a WT and Neuro2a-shuPAR cells as verified by RT-qPCR; Figure S4. Original western blot images. Table S1: Murine primers used in the study for RT-qPCR.
