# Supplementary material for: Urokinase Receptor uPAR Downregulation in Neuroblastoma Leads to Dormancy, Chemoresistance and Metastasis
Source: Cancers (Basel). 2022 Feb 16;14(4):994. doi: 10.3390/cancers14040994 (PMC8870350; doi:10.3390/cancers14040994)
Supplement: Supplementary file 1 [file cancers-14-00994-s001.zip › cancers-1562608-supplementary.pdf]

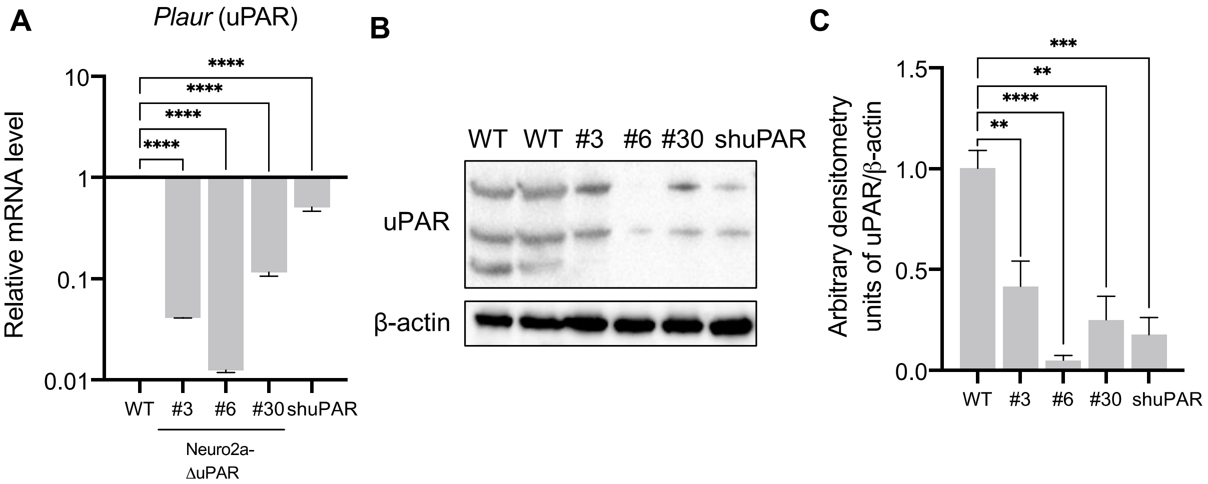

Figure S1. Plaur (uPAR) expression in Neuro2a cells analyzed by RT-qPCR. (A) The mRNA level of *Plaur* in Neuro2a was evaluated using RT-qPCR; data were normalized to *Actb* expression as a housekeeping gene. Data are presented on log10 scale as individual values, mean ± SEM. (B) Western blot analysis of uPAR content in Neuro2a cells. β-actin was used as loading control. A typical result from three independent experiments is presented. (C) Densitometry analysis of uPAR content normalized to β-actin. Data are presented as mean ± SEM. WT – control Neuro2a cells; #3, #6, and #30 – uPAR-deficient clones of Neuro2a cells; Neuro2a-shuPAR – cells transfected with shRNA to suppress uPAR. ** $P < 0.01$, *** $P < 0.001$, **** $P < 0.0001$ compared with WT (ANOVA, Dunnett's post hoc test).

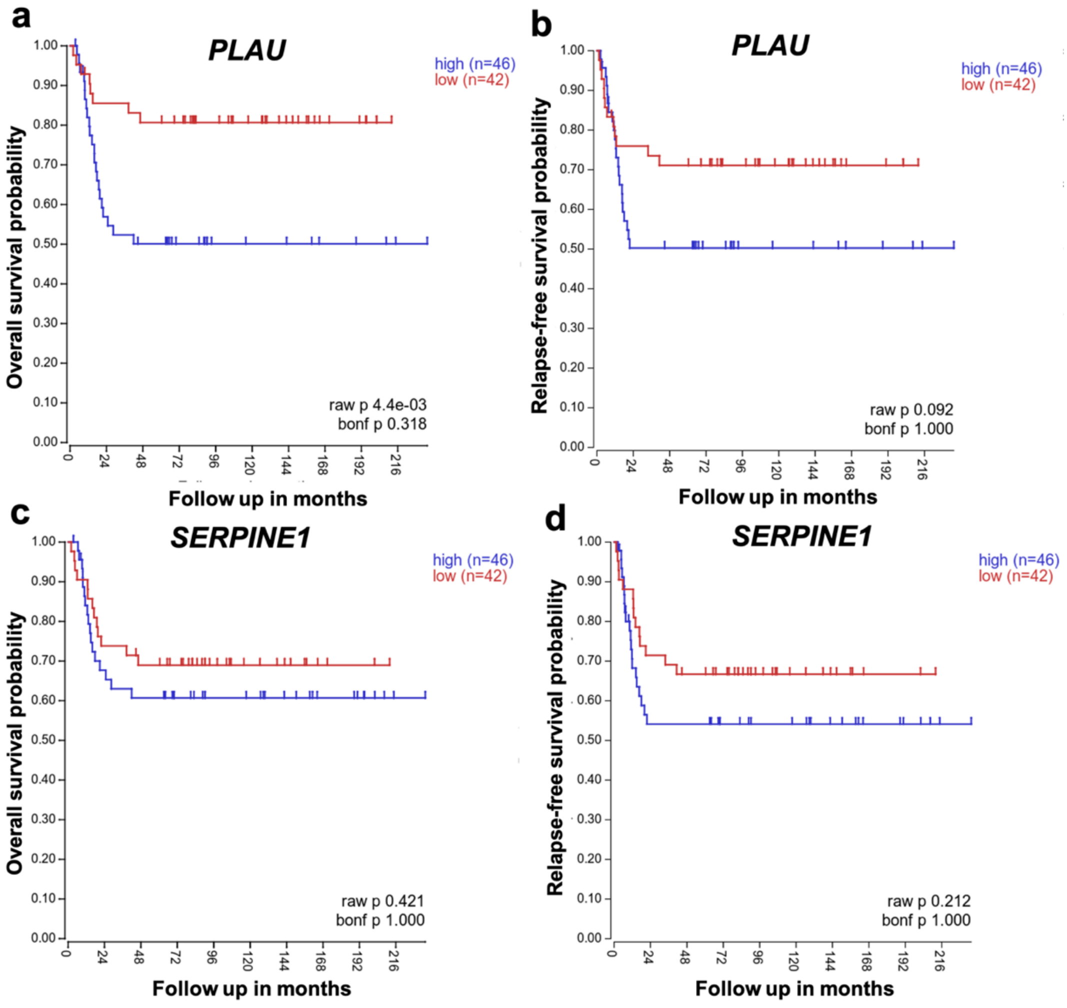

Figure S2. Overall and relapse-free survival of human neuroblastoma patients stratified by uPA and PAI-1 expression. (A) Overall survival of human neuroblastoma patients stratified by low (red) and high (blue) expression of PLAU. (B) Relapse-free survival of human neuroblastoma patients stratified by low (red) and high (blue) expression of PLAU. (C) Overall survival of human neuroblastoma patients stratified by low (red) and high (blue) expression of SERPINE1. (D) Relapse-free survival of human neuroblastoma patients stratified by low (red) and high (blue) expression of SERPINE1. Kaplan-Meier survival curves were generated from Versteeg cohort 26 data, NCBI GEO accession GSE16476, and compared using R2 database (http://r2.amc.nl). All adjusted P values > 0.05.

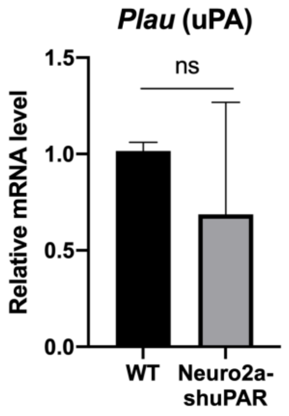

Figure S3. *Plau* (uPA) mRNA expression in Neuro2a WT and Neuro2a-shuPAR cells as verified by RT-qPCR. The mRNA level of Plau in Neuro2a was evaluated using RT-qPCR; data were normalized to *Actb* expression as a housekeeping gene. Data are presented as mean ± SEM, ns – non-significant (t-test).

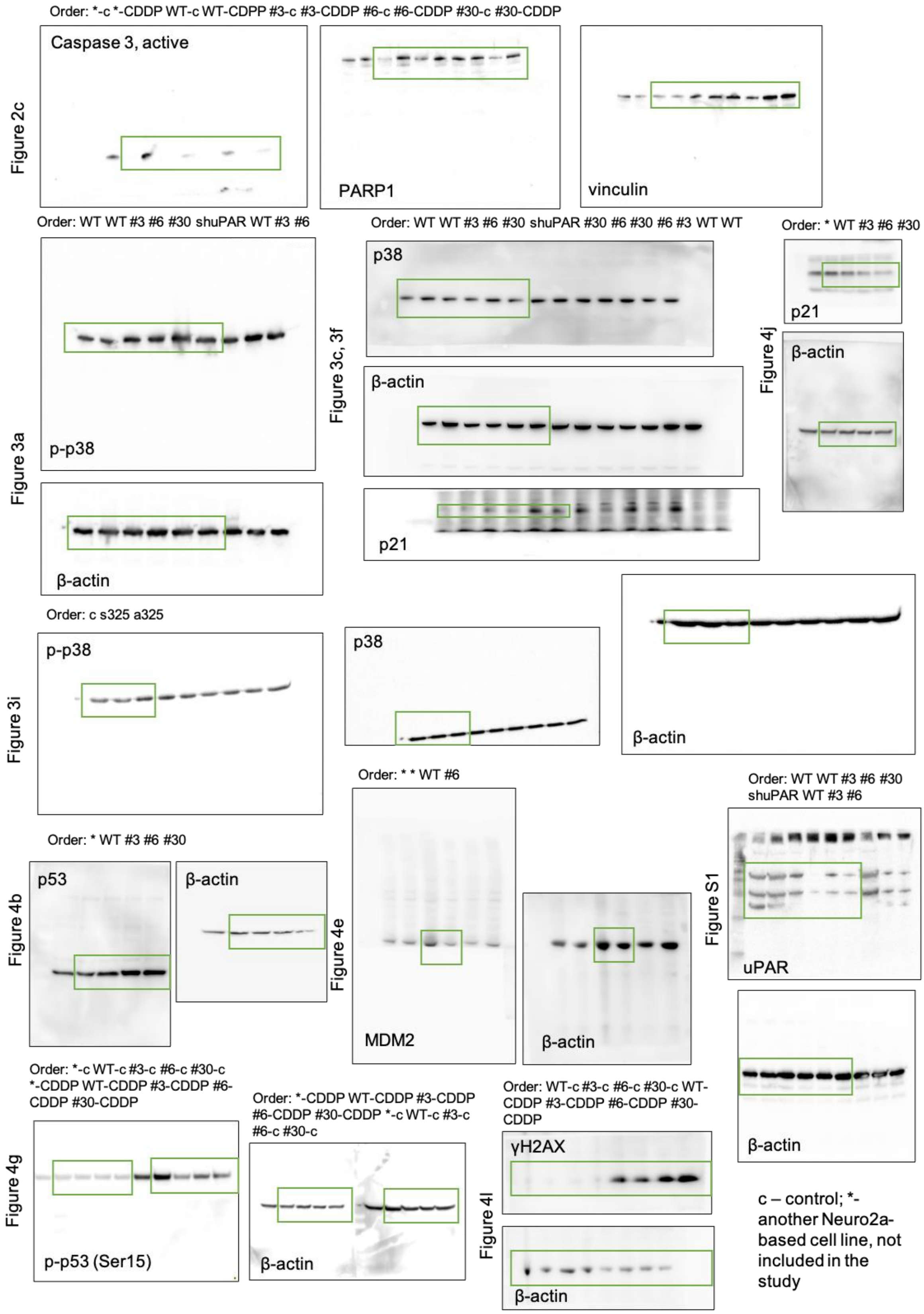

Figure S4. Original western blot images. Green rectangles indicate cropped regions.

Table S1. Murine primers used in the study for RT-qPCR.

Primer	Sequence 5'->3'
Plau forward	ATGGAAATGGTGACTCTTACCGA
Plau reverse	TGGGCATTGTAGGGTTTCTGA
Plaur forward	CGCCACAAACCTCTGCAAC
Plaur reverse	CTCTGTAGGATAGCGGCATTG
Trp53 forward	CCCCTGTCATCTTTTGTCCCT
Trp53 reverse	AGCTGGCAGAATAGCTTATTGAG
Cdkn1a forward	CGAGAACGGTGGAACTTTGAC
Cdkn1a reverse	CAGGGCTCAGGTAGACCTTG
Mdm2 forward	GGATCTTGACGATGGCGTAAG
Mdm2 reverse	AGGCTGTAATCTTCCGAGTCC
Actb forward	AGTGTGACGTTGACATCCGTA
Actb reverse	GCCAGAGCAGTAATCTCCTTCT
